# PD-L1 Expression in Monocytes Correlates with Bacterial Burden and Treatment Outcomes in Active Pulmonary Tuberculosis

**DOI:** 10.3390/ijms23031619

**Published:** 2022-01-30

**Authors:** Sheng-Wei Pan, Chin-Chung Shu, Jhong-Ru Huang, Chang-Ching Lee, Yen-Han Tseng, Jung-Jyh Hung, Po-Kuei Hsu, Nien-Jung Chen, Wei-Juin Su, Jia-Yih Feng, Yuh-Min Chen

**Affiliations:** 1 Department of Chest Medicine, Taipei Veterans General Hospital, Taipei 112, Taiwan; swpan25@gmail.com (S.-W.P.); pcipur@gmail.com (J.-R.H.); Johnny7742@hotmail.com (C.-C.L.); yenhan213@hotmail.com (Y.-H.T.); ymchen@vghtpe.gov.tw (Y.-M.C.); 2 School of Medicine, National Yang Ming Chiao Tung University, Taipei 112, Taiwan; jjhung2@vghtpe.gov.tw (J.-J.H.); pkhsu@vghtpe.gov.tw (P.-K.H.); wjsu.mail@gmail.com (W.-J.S.); 3 Institute of Public Health, National Yang Ming Chiao Tung University, Taipei 112, Taiwan; 4 Department of Internal Medicine, National Taiwan University Hospital, Taipei 110, Taiwan; stree139@yahoo.com.tw; 5 College of Medicine, National Taiwan University, Taipei 110, Taiwan; 6 Division of Thoracic Surgery, Department of Surgery, Taipei Veterans General Hospital, Taipei 112, Taiwan; 7 Institute of Microbiology and Immunology, School of Life Sciences, National Yang Ming Chiao Tung University, Taipei 112, Taiwan; njchen@ms.ym.edu.tw; 8 Division of Chest Medicine, China Medical University Hospital, Taipei Branch, Taipei 114, Taiwan; 9 Institute of Emergency and Critical Care Medicine, National Yang Ming Chiao Tung University, Taipei 112, Taiwan

**Keywords:** PD-1, PD-L1, pulmonary tuberculosis, macrophages, treatment outcomes

## Abstract

The PD-1/PD-L1 pathway is critical in T cell biology; however, the role of the PD-1/PD-L1 pathway in clinical characteristics and treatment outcomes in pulmonary tuberculosis (PTB) patients is unclear. We prospectively enrolled PTB, latent TB infection (LTBI), and non-TB, non-LTBI subjects. The expression of PD-1/PD-L1 on peripheral blood mononuclear cells (PBMCs) was measured and correlated with clinical characteristics and treatment outcomes in PTB patients. Immunohistochemistry and immunofluorescence were used to visualize PD-1/PD-L1-expressing cells in lung tissues from PTB patients and from murine with heat-killed MTB (HK-MTB) treatment. A total of 76 PTB, 40 LTBI, and 28 non-TB, non-LTBI subjects were enrolled. The expression of PD-1 on CD4+ T cells and PD-L1 on CD14+ monocytes was significantly higher in PTB cases than non-TB subjects. PTB patients with sputum smear/culture unconversion displayed higher PD-L1 expression on monocytes. PD-L1-expressing macrophages were identified in lung tissue from PTB patients, and co-localized with macrophages in murine lung tissues. *Mycobacterium tuberculosis* (MTB) whole cell lysate/EsxA stimulation of human and mouse macrophages demonstrated increased PD-L1 expression. In conclusion, increased expression of PD-L1 on monocytes in PTB patients correlated with higher bacterial burden and worse treatment outcomes. The findings suggest the involvement of the PD-1/PD-L1 pathway in MTB-related immune responses.

## 1. Introduction

Tuberculosis (TB) is an aerosol-transmitted infectious disease with a high rate of morbidity and mortality. Both innate and adaptive immunity are involved in host defense mechanisms against *Mycobacterium tuberculosis* (MTB) in human lungs. Macrophages are crucial innate cells. In particular, alveolar macrophages are the first line of defense against MTB [1]. CD4+ and CD8+ T cells are key effector cells in adaptive immune responses. Both release pro-inflammatory cytokines and produce cytotoxic molecules to quell MTB infection [2,3,4].

The dysfunction of MTB-specific and non-specific CD4+ and CD8+ T cells (i.e., T cell exhaustion) has been described in active TB patients, and is associated with worse treatment outcomes [5,6,7]. Programmed death 1 (PD-1) receptor protein is typically expressed by activated lymphocytes, and acts as an immune checkpoint due to its suppression of T cell-mediated immune responses [8,9,10]. Programmed death-ligand 1 (PD-L1) is the PD-1 ligand expressed on various cells, including lymphocytes, monocytes, macrophages, and dendritic cells (DCs). PD-L1 is typically upregulated with activation [11]. PD-1/PD-L1 pathway activation is reportedly involved in T cell dysfunction during tumorigenesis and chronic viral infections [12,13,14].

The role of the PD-1/PD-L1 pathway in the pathogenesis of MTB infection remains controversial. The upregulation of PD-1 expression in tissues and peripheral blood has been reported in humans and animals with MTB infection [15,16]. Blockade of PD-1/PD-L1 therapies can enhance TB-specific T cell responses in active TB patients [17,18]. However, PD-1 knock-out mice display enhanced susceptibility to MTB infection, and are associated with increased bacterial load and reduced survival [19,20,21]. Reactivation of TB is commonly reported in cancer patients receiving PD-1/PD-L1 blockade therapy [18,22]. These findings suggest the multifaceted roles of PD-1/PD-L1 in the progression of MTB infection.

The interaction between innate and adaptive immunity plays a pivotal role in host immune responses against MTB. The expression of PD-L1 in antigen-presenting cells upon MTB infection may bind PD-1 on lymphocytes and activate the downstream pathway, which can lead to the immune exhaustion of lymphocytes [23]. Increased expression of PD-1 and PD-L1 have been reported as important characteristics in TB infection [24,25]. This could be used by MTB bacilli to suppress host immune responses and facilitate MTB infection. We hypothesize that PD-1 and PD-L1 expression on host immune cells may be correlated with bacteria burden and associated with treatment outcomes in active TB patients. The present study aimed to explore the characteristics of PD-1/PD-L1 expression in peripheral blood mononuclear cells (PBMCs) of active TB patients, and the association of such expression with clinical presentation and treatment outcomes. The expression of PD-1/PD-L1 in lung tissues from active TB patients and mice treated with MTB stimulation were also investigated.

## 2. Results

### 2.1. Patient Characteristics

During the study period, a total of 76 active pulmonary TB patients were prospectively enrolled. Forty LTBI cases and 28 non-TB, non-LTBI subjects were enrolled as comparators. Demographic characteristics are shown in Table 1. Active TB patients were older than non-TB subjects and had fewer lymphocyte counts than non-TB, non-LTBI individuals (Table 1). Monocyte counts were comparable between active TB and non-TB cases. Lymphocyte counts were lower in active TB patients, compared with non-TB, non-LTBI subjects.

### 2.2. PD-1 and PD-L1 Expression on PBMCs from Pulmonary TB Patients

**Figure 1:**
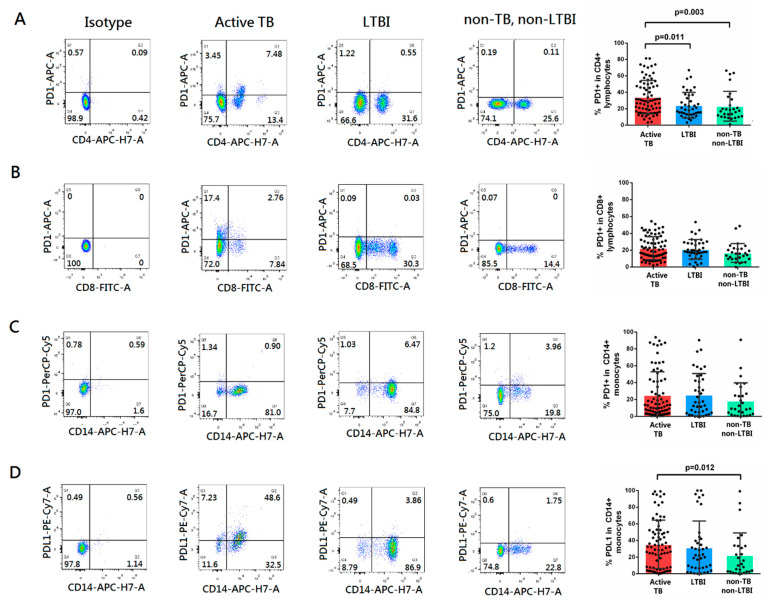
Representative flow cytometry data for PD-1 and PD-L1 expressed in peripheral blood mononuclear cells from individuals with PTB (*n* = 76), LTBI (*n* = 40), and non-TB, non-LTBI subjects (*n* = 28). The proportion of PD-1 in (**A**) CD4+ lymphocytes, (**B**) CD8+ lymphocytes, (**C**) CD14+ monocytes, and (**D**) PD-L1 in CD14+ monocytes are presented. Dot plot of distributions of PD-1 and PD-L1 positive cells in PTB, LTBI, and non-TB, non-LTBI individuals are also shown. Data are presented as individual measurements with mean values. Differences between groups were compared using the Mann-Whitney U test. PTB, pulmonary tuberculosis; LTBI, latent tuberculosis infection; PD-1, programmed death-1; PD-L1, programmed death-ligand 1.

The expression of PD-1 and PD-L1 on CD4+ T cells, CD8+ T cells, and CD14+ monocytes in PBMCs from pulmonary TB patients, LTBI cases, and non-TB, non-LTBI individuals was measured by flow cytometry. PD-1 expression was significantly higher on CD4+ T cells in pulmonary TB patients, compared with LTBI cases (*p* = 0.011) and non-TB, non-LTBI individuals (Figure 1, *p* = 0.003). The expression of PD-L1 on CD14+ monocytes was significantly higher in pulmonary TB patients compared with non-TB, non-LTBI individuals (*p* = 0.012). The expression of PD-1 on CD8+ T cells and CD14+ monocytes were similar between enrolled cases.

### 2.3. Differences of PD-1 and PD-L1 Expression in Pulmonary TB Patients with Various Clinical Presentations and Treatment Outcomes

**Figure 2:**
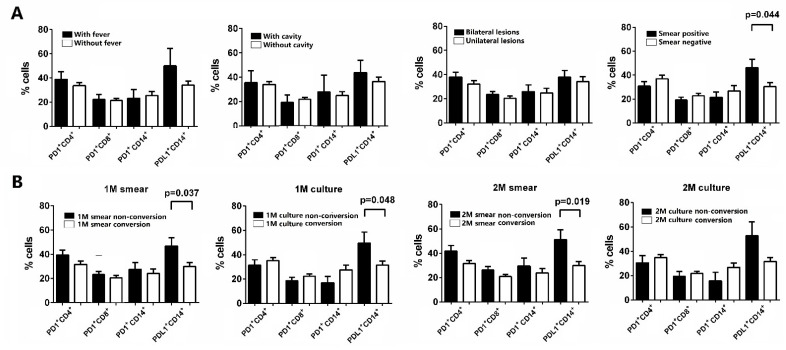
Proportions of PD-1 and PD-L1 expressing cells in peripheral blood mononuclear cells of PTB patients with (**A**) various clinical presentations and (**B**) different treatment outcomes. The data in bar charts are mean values. Error bars are standard errors. Differences between groups were compared using the Mann–Whitney U test. PTB, pulmonary tuberculosis; PD-1, programmed death-1; PD-L1, programmed death-ligand 1.

We further analyzed the expression of PD-1 and PD-L1 in pulmonary TB patients with various clinical and radiological presentations, including presence of fever, cavitary lesions and bilateral involvement in chest plain films, and positive smear microscopy results. The expression of PD-L1 on CD14+ monocytes was significantly higher in smear-positive active TB patients, compared with smear-negative active TB patients (Figure 2A). A trend toward higher PD-L1 expression on CD14+ monocytes in active TB patients with fever was evident compared to those without fever.

We also compared the expression of PD-1 and PD-L1 in pulmonary TB patients with different treatment responses. Expressions of PD-L1 on CD14+ monocytes in pulmonary TB patients before treatment were significantly lower in cases with 1-month or 2-month smear conversion, and 1-month culture conversion compared to those without smear/culture conversion (Figure 2B). The expression of PD-1 on CD4+ and CD8+ T cells, and PD-1 on CD14+ monocytes before treatment was comparable between active TB patients with and without 1- and 2-month smear/culture conversion.

### 2.4. Change of PD-1 and PD-L1 Expression after Anti-TB Treatment

**Figure 3:**
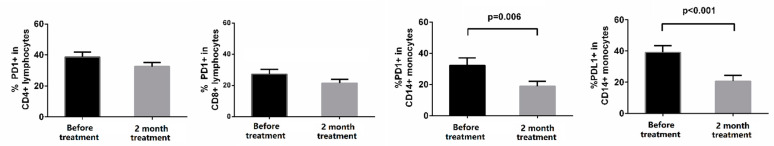
Proportions of PD-1 and PD-L1 expressing cells in peripheral blood mononuclear cells of PTB patients, before and after 2 months of TB treatment. The data in bar charts are mean values. Error bars are standard errors. The data were compared using the Mann–Whitney U test. PTB, pulmonary tuberculosis; PD-1, programmed death-1; PD-L1, programmed death-ligand 1.

Among the 76 pulmonary TB patients, PBMCs were collected again in 41 after 2 months of anti-TB treatment. The results of the comparisons of PD-1 and PD-L1 expression on CD4+ and CD8+ T cells, and CD14+ monocytes are shown in Figure 3. Expression of PD-1 and PD-L1 on CD14+ monocytes significantly declined after 2 months of anti-TB treatment (PD-1, 32.2 ± 31.2% vs. 18.9 ± 16.98%, *p* = 0.006; PD-L1, 39.2 ± 27.9% vs. 20.7 ± 23.4%, *p* < 0.001). There was no significant difference in PD-1 expression on CD4+ and CD8+ T cells, before or after 2 months of anti-TB treatment.

### 2.5. PD-1 and PD-L1 Expression on Human and Mice Macrophages

**Figure 4:**
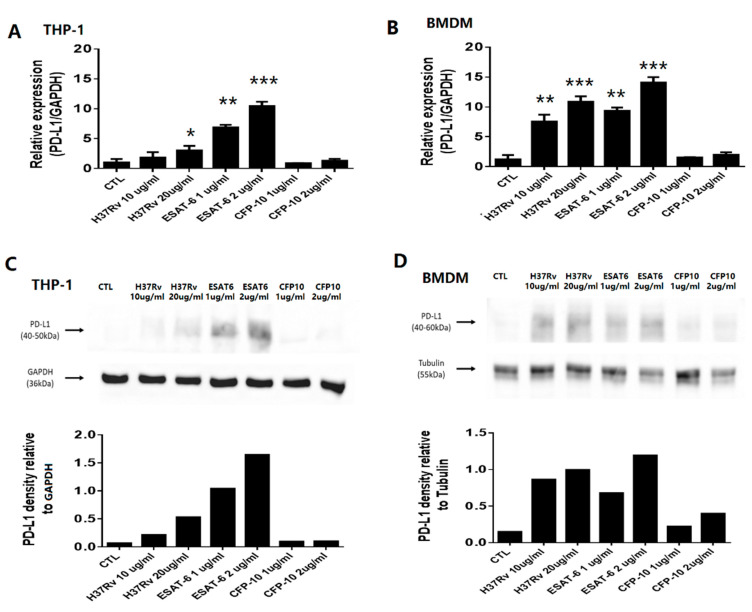
PD-L1 production and gene expression by THP-1 cells and BMDMs isolated from wild-type mice. THP-1 cells and BMDMs were treated with H37Rv whole cell lysate, EsxA, and CFP-10. Gene expression of *PD-L1* from (**A**) THP-1 cells and (**B**) BMDMs significantly increased after stimulation with H37Rv whole cell lysate and EsxA, but not in CFP-10. Western blot assay of PD-L1 and densitometry in (**C**) THP-1 cells and (**D**) BMDMs demonstrated findings similar to PD-L1 gene expression. The concentration of H37Rv whole cell lysate was 10 and 20 μg/mL. The concentration of EsxA and CFP-10 was 1 and 2 μg/mL. Data are presented as the mean ± standard deviation (SD) for each group. PD-L1, programmed death-ligand 1. BMDM, bone marrow derived macrophage. *: *p* < 0.05; **: *p* < 0.01; ***: *p* < 0.001.

Considering the association between PD-L1 expression on CD14+ monocytes and clinical presentations and treatment outcomes in active TB patients, we measured the expression of the *PD-L1* gene in THP-1 cells and mice BMDMs. A significantly increased expression of the *PD-L1* gene in THP-1 cells was evident after stimulation with H37Rv whole cell lysate (10 and 20 µg/mL) and EsxA protein (1 and 2 µg/mL), but not in CFP-10 stimulation (Figure 4A). In BMDMs, the increased expression of *PD-L1* gene was also observed after stimulation with H37Rv whole cell lysate (1 and 10 µg/mL) and EsxA protein (1 and 2 µg/mL), but not CFP-10 protein (Figure 4B). Increased production of PD-L1 protein in THP-1 cells and BMDMs after stimulation with H37Rv whole cell lysate and EsxA protein was also revealed by western blotting (Figure 4C,D).

### 2.6. Detection of PD-L1-Expressed Macrophages in Lung Tissues Exposed to MTB

**Figure 5:**
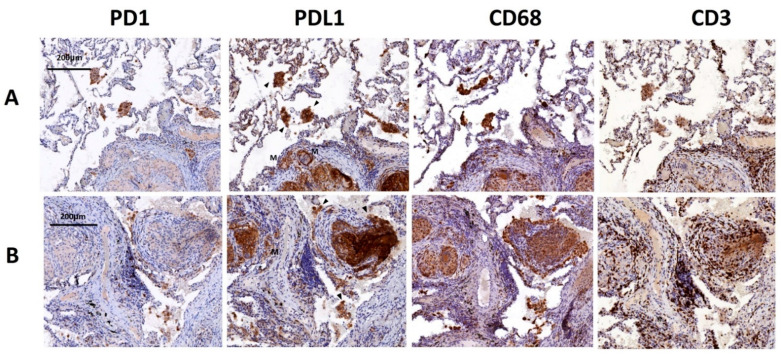
Immunohistochemistry (IHC) staining of lung tissues from two patients with pulmonary TB. PD-1 and PD-L1 positive cells in lung specimens from patients with pulmonary TB were demonstrated. IHC staining using an anti-PD-L1 antibody showed that PD-L1 positive cells were localized in the (**A**) alveolar air spaces (arrow heads), (**B**) granulomas and multinuclear giant cells. IHC staining using an anti-CD68 antibody confirmed that PD-L1-positive cells were mostly macrophages. M indicates multinuclear giant cells. Arrowheads indicate PD-L1-positive cells. PD-1, programmed death-1; PD-L1, programmed death-ligand 1.

IHC analysis was done in lung tissues from two pulmonary TB patients, which demonstrated granulomatous inflammation and inflammatory cell infiltration in alveolar spaces. As shown in Figure 5, IHC staining identified PD-L1 strongly positive cells within granulation tissues and alveolar spaces. These cells were mostly positive for CD68 with a few positive for CD3, indicating that these cells were mostly macrophages. In contrast, PD-1 expression was much less than PD-L1 in lung tissues.

**Figure 6:**
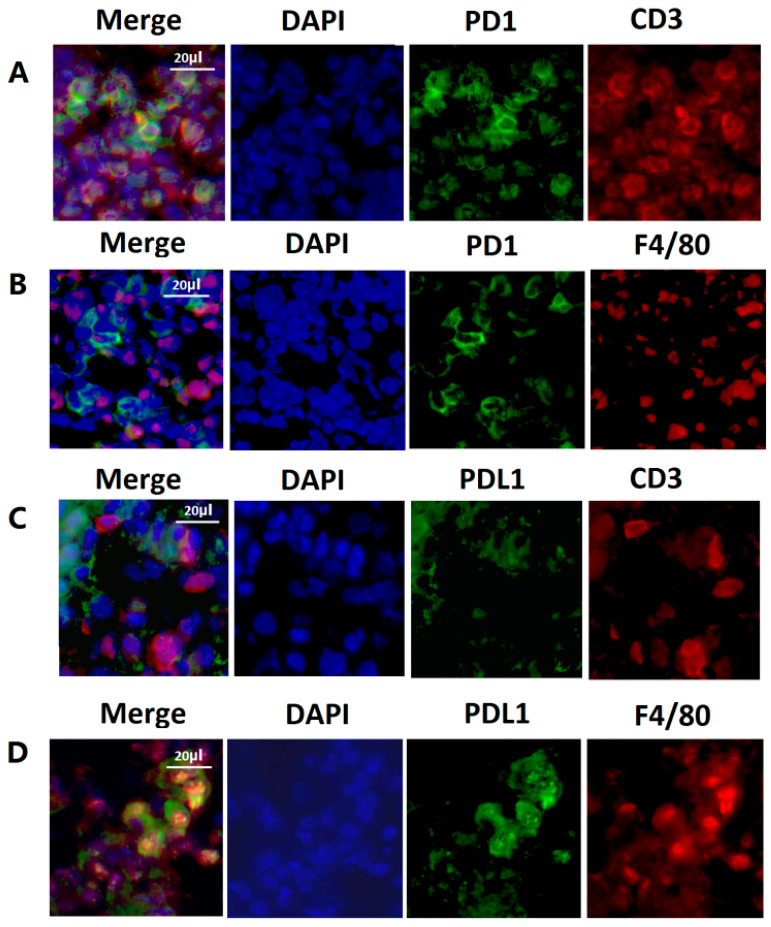
Immunofluorescence staining of PD-1 and PD-L1 in lung tissues harvested from C57BL/6 mice treated with intratracheal instillation of HKMTB for 96 h. The expression of PD-1 (green) overlapped with (**A**) CD3+ lymphocytes (red) but not (**B**) F4/80+ macrophages (red). The expression of PD-L1 (green) overlapped with (**D**) F4/80+ macrophages (red), but not (**C**) CD3+ lymphocytes (red). PD-1, programmed death-1; PD-L1, programmed death-ligand 1.

Lung tissues from mice with intratracheal instillation of HKMTB demonstrated inflammatory cell infiltration. PD-1 and PD-L1 expressing cells were observed in lung tissues from mice with HKMTB instillation. In the merged pictures, the immunoreactivity toward PD-1 antibody colocalized with the staining of CD3 (Figure 6A), and the immunoreactivity toward PD-L1 antibody colocalized with the staining of F4/80 (Figure 6D). The findings suggested that most PD-1-expressed cells were lymphocytes, and most PD-L1-expressed cells were macrophages.

## 3. Discussion

This prospective observational study enrolled pulmonary TB patients and investigated the expression of PD-1 and PD-L1 on T cells and monocytes in PBMCs. The increased expression of PD-1 on CD4+ T cells and PD-L1 on CD14+ monocytes was evident in pulmonary TB patients, and the increased expression of PD-L1 on CD14+ monocytes was associated with higher bacterial burden and delayed smear/culture conversion. The decreased expression of PD-L1 on CD14+ monocytes was evident after anti-TB treatment. The findings were verified in cell and animal models. After MTB-related stimulation, human macrophages and murine BMDMs demonstrated the increased expression of PD-L1. Finally, PD-L1-expressed immune cells were identified in lung tissues from pulmonary TB patients and mice with intratracheal instillation of HKMTB. IHC and IF analyses indicated the increased expression of PD-L1 in macrophages in lung tissues.

Several previous human and animal studies have suggested that the PD-1/PD-L1 pathway is actively involved in the tuberculosis pathogenesis. Increased numbers of PD-1-expressing CD4+ and/or CD8+ T cells in PBMCs and the pleural fluid of active TB patients have been described, and the expression is decreased after effective anti-TB treatment [26,27,28]. The blockade of PD-1/PD-L1 signaling in vitro can augment inflammatory cytokine production in CD4+ T cells, and induce cytotoxicity in CD8+ T cells [27,29]. Similar findings were also reported in monkeys [15]. In the present study, PD-1 expression was similarly increased in CD4+ T cells compared to non-TB and non-LTBI individuals. However, PD-1 expression did not differ on CD4+ and CD8+ T cells in active TB patients with various clinical presentations, including sputum smear status. A major difference between the previous and present studies is that some previous studies evaluated MTB-specific CD4+ and CD8+ T cells, not non-MTB-specific T cells [27,28]. However, the characteristics of non-TB-specific immune responses remain a critical issue that deserves evaluation [7]. The immune characteristics of T cells may be different in peripheral blood and lesion sites, such as pleural fluids, and such differences could contribute to the discrepancy between prior findings and our results.

The expression of PD-L1 on CD14+ monocytes in active TB patients has been unclear. Previous studies have reported the increased expression PD-L1 on CD14+ monocytes in PBMCs and pleural fluids of active TB patients compared with non-TB subjects [16,30]. However, the association between PD-L1 expression on monocytes and clinical characteristics of active TB patients were not examined. In the present study, in addition to increased expression of PD-L1 on monocytes in active TB patients, the expression of PD-L1 on CD14+ monocytes was significantly higher in smear-positive pulmonary TB patients and those with delayed sputum smear/culture conversion. The expression of PD-L1 on CD14+ monocytes significantly declined after anti-TB treatment. Our findings suggest that the expression of PD-L1 on CD14+ monocytes is closely correlated with disease severity and treatment responses, as compared to PD-1 expression on T cells in TB patients. Thus, PD-L1 expression on monocytes may also be important in MTB-related immune reactions.

Information regarding the expression of PD-1 and PD-L1 in T cells and monocytes stimulated with TB-related antigens is limited. Day et al. used the EsxA/CFP-10 peptide pool to treat PBMCs isolated from active TB patients and reported the upregulation of PD-1 on MTB-specific CD4+ T cells [27]. The increased expression of PD-L1 in tumor-associated macrophages has been widely reported in various types of malignancies and serves as an important mechanism of tumorigenesis [31,32]. However, the role of MTB-related stimulation in PD-L1 expression on macrophages and its association with MTB infection remains uncertain. In the present study, both H37Rv whole cell lysate and EsxA induced PD-L1 gene expression and protein production, in both human macrophage cells and mouse BMDMs. In contrast, the response was relatively low upon CFP-10 stimulation. To our knowledge, this is the first report of increased PD-L1 expression on macrophages stimulated with MTB-specific proteins, especially EsxA. EsxA is an MTB-specific protein associated with mycobacterial virulence. It facilitates MTB replication and transmission inside macrophages [33]. EsxA also has immunomodulatory effects, interacting with the Toll-like receptor (TLR)-2 [34]. Our cell experimental findings suggest that EsxA may exert its immunomodulation effect through the PD-1/PD-L1 pathway in macrophages, which is pivotal in innate immunity against MTB. In addition to increased PD-1 on T cells, the expression of PD-L1 on macrophages can further amplify the PD-1/PD-L1 pathway through crosstalk between T cells and macrophages.

We visualized the PD-1- and PD-L1-expressing immune cells in lung tissues by IHC and IF. Abundant PD-L1 expressing macrophages were identified in granuloma and alveolar spaces in lung tissues obtained from pulmonary TB patients. IF analysis confirmed the co-localization of PD-L1 and macrophages in lung tissues from mice with the instillation of HKMTB. This is the first study to use IHC and IF analyses to visualize PD-1 and PD-L1-expressing inflammatory cells in lung tissues with MTB infection/stimulation. The findings provide direct pathological evidence to indicate the involvement of PD-L1-expressing macrophages in MTB-induced pulmonary infection. However, whether activation of the PD-1/PD-L1 pathway is detrimental or beneficial to pulmonary TB remains unclear. Although the PD-1/PD-L1 pathway is a negative regulator of Th-1-type immunity of T cells and is regarded as a T cell inhibitory molecule, it is also important in maintaining the homeostasis of immune responses [35]. The PD-1/PD-L1 blockade in mice displayed enhanced susceptibility to MTB, increased necrotic reaction in lung tissue, and increased mortality [19,20,21]. The reactivation of TB following treatment with PD-1/PD-L1 inhibitors is not a rare event in cancer patients [36,37]. These reports indicate the critical role of the PD-1/PD-L1 pathway in maintaining the balance of immune responses. Both over-expression and under-expression of PD-1/PD-L1 could be harmful to the host.

This study had several limitations. The expression of PD-1 and PD-L1 was evaluated in non-TB-specific T cells and monocytes, not MTB-specific immune cells. However, we previously demonstrated the strong association between non-TB-specific immune responses and treatment outcomes in active TB patients, suggesting that PD-1/PD-L1 expression on non-MTB-specific immune cells should not be overlooked. Second, this study measured the expression of PD-1/PD-L1 in immune cells from peripheral blood, not at lesion sites, such as pleural fluid or bronchoalveolar lavage fluid. The characteristics of PD-1/PD-L1 expression on immune cells from peripheral blood and lesion sites may not be identical. Finally, we used intratracheal instillation of HKMTB in mice to mimic MTB infection. Further studies with live MTB infection are needed to verify our findings.

## 4. Materials and Methods

### 4.1. Patients and Settings

This prospective observational study was conducted in two tertiary medical centers in northern Taiwan. Newly diagnosed, culture-proven, pulmonary TB patients from August 2017 to July 2020 were eligible for enrollment. We also enrolled individuals with latent TB infection (LTBI) and non-TB, non-LTBI subjects as comparators. Demographic characteristics of each enrolled patient were obtained during an enrollment interview. Radiographic presentations of the active TB patients were reviewed by the in-charge doctors, and the presence of cavitary lesion were recorded accordingly. This study was approved by the Institutional Review Board of participating hospitals (IRB number 2017-12-004CC A, 07/02/2017; 201711060RIND, 05/01/2018). Written informed consent was obtained from each participant or their authorized representative(s) before enrollment. Details are shown in Supplementary material.

### 4.2. Expression of PD-1 and PD-L1 on Peripheral Blood Mononuclear Cells (PBMCs)

Peripheral blood samples were collected from the participants on the day of enrollment. Fluorescently-conjugated human monoclonal antibodies were used to determine the expression of PD-1 and PD-L1 on CD4+ T cells, CD8+ T cells, and CD14+ monocytes. The expression of PD-1 and PD-L1 were measured using flow cytometry (FACSVerse; BD Biosciences, San Jose, CA, USA).

### 4.3. Clinical Features and Treatment Outcomes of Active TB Patients

The expression of PD-1 and PD-L1 on PBMCs was compared among active TB patients with different clinical presentations and treatment outcomes. The clinical presentations analyzed included the presence of fever ≥ 38 °C and the presence of cavity and bilateral involvement in chest radiography. Bacterial burden was evaluated by sputum smear microscopy. The treatment outcomes analyzed in the present study included 1- and 2-month smear/culture conversion.

### 4.4. Preparation of Human and Mouse Macrophages

The findings of PD-L1 expression on monocytes of active TB patients were verified using THP-1 human leukemia cells (TIB202; ATCC; Manassas, VA, USA) and bone marrow-derived macrophages (BMDMs) from the femur and tibia of C57BL/6J mice. The animal experiment was approved by the institutional animal ethical approval of National Yang Ming University (1070707, 31 July 2018). Details are shown in Supplementary material.

### 4.5. MTB-Related Materials and In Vitro Stimulation

MTB-related materials included MTB whole cell lysates (M. tuberculosis, Strain H37Rv, Whole Cell Lysate, NR-14822), recombinant EsxA protein (Recombinant Protein Reference Standard, NR-49424), and recombinant CFP-10 protein (Recombinant Protein Reference Standard, NR- 49425) (all from BEI Resources; Manassas, VA, USA). Both THP-1 cells and BMDMs of mice were treated with MTB whole cell lysates (10 and 20 µg/mL), EsxA protein (1 and 2 µg/mL), and CFP-10 (1 and 2 µg/mL) for 6 h for RNA isolation and for 24 h for Western blot assay. Details are shown in Supplementary material.

### 4.6. Lung Tissues from Active TB Patients and Mice

Two patients with active pulmonary TB and available lung tissues obtained from surgical procedures were prospectively enrolled. Both lung specimens showed necrotizing granulomatous inflammation with the presence of acid-fast bacilli and were culture positive for MTB. Their lung tissues were used for immunohistochemistry (IHC) analysis.

To obtain lung tissues from mice stimulated with MTB, intratracheal instillation was performed in wild-type C57BL/6J mice, as previous described [38]. After anesthesia by intraperitoneal injection of avertin, 50 µL of a 0.003 µg/µL preparation of diluted heat-killed MTB (HKMTB) H37Ra (Difco Laboratories, Detroit, MI, USA) was instilled into the trachea of mice at a dose of 0.006 µg/g. The mice were sacrificed 96 h after instillation. After thoracotomy, the lungs were prepared for microscopic evaluation and immunofluorescence (IF) assays.

### 4.7. IHC and IF Analysis

To identify PD-1 and PD-L1 expressing cells, lung specimens obtained from two active pulmonary TB patients analyzed by IHC. IF was performed to evaluate PD-1 and PD-L1 expression in lung specimens from mice following the intratracheal instillation of HKMTB. To obtain lung tissue, perfusion was performed by injecting 5 mL cold PBS through the right ventricle of the heart before embedding. Both IHC and IF analysis were performed in the core facility of Taipei Veterans General Hospital. Details are shown in Supplementary material.

### 4.8. Statistical Analysis

Inter-group differences were analyzed using the Mann-Whitney U test for numerical variables, and Fisher’s exact test was used for categorical variables. Statistical significance was set at *p* < 0.05. All analyses were performed using SPSS version 25.0 (Chicago, IL, USA).

## 5. Conclusions

This prospective observational study evaluated the characteristics of PD-1/PD-L1 expression on T cells and monocytes in PBMCs from active TB patients, and used in vitro and in vivo (mouse) models to verify the findings. Active TB patients had an increased expression CD4+ T cells and PD-L1 on CD14+ monocytes. The higher expression of PD-L1 on CD14+ monocytes was associated with higher bacterial burden and worse treatment responses. Experiments in in vitro and in vivo models demonstrated that MTB-related stimulation, especially EsxA, upregulated PD-L1 expression and PD-L1 protein production in macrophages. IHC and IF analyses confirmed the co-localization of PD-L1 and macrophages in lung tissue. Our findings suggest that PD-L1 expression on monocytes in peripheral blood and on macrophages in lung tissue is actively involved in MTB-induced inflammation. Further studies are warranted to dissect the exact role of PD-L1 on monocytes/macrophages in the pathogenesis of MTB infection.

## Figures and Tables

**Table 1:** Demographic characteristics of patients with PTB, LTBI, and non-TB, non-LTBI subjects ^a,b^.

	PTB, *n* = 76	LTBI, *n* = 40	Non-TB, Non-LTBI, *n* = 28
Mean age (SD)	65.0 (18.7)	47.3 (17.8) ***	42.1 (25.1) **
Male sex	50 (65.8%)	25 (62.5%)	15 (53.6%)
BMI (SD)	21.7 (4.0)	22.6 (3.4)	20.6 (3.7)
Smoking history	29 (38.2%)	18 (45%)	5 (17.9%) *
BCG vaccination	53 (69.7%)	29 (72.5%)	22 (78.6%)
Prior TB treatment history	6 (7.9%)	2 (5.0%)	2 (7.1%)
Diabetes	13 (17.1%)	4 (10%)	4 (14.3%) *
Renal insufficiency	2 (2.6%)	4 (10%)	0
COPD	1 (1.3%)	4 (10%)	0
Malignancy	6 (7.9%)	5 (12.5%)	0
Post gastrectomy	1 (1.3%)	0	0
Cell count (Median, IQR)			
Neutrophils (×10^9^ per L)	3878 (2617–4750)	4097 (2496–5219)	4197 (2967–5112)
Lymphocyte (×10^9^ per L)	1432 (823–1765)	1666 (1141–2140)	1895 (1401–2326) **
Monocytes (×10^9^ per L)	466 (297–593)	454 (325–548)	492 (292–518)

^a^ Data are presented as mean ± SD or *n*(%) unless otherwise stated. ^b^ * *p* < 0.05; ** *p* < 0.01; *** *p* < 0.001 BMI, body mass index; BCG, bacillus Calmette–Guérin; PTB, pulmonary tuberculosis; TB tuberculosis; LTBI, latent tuberculosis infection; COPD, chronic obstructive pulmonary disease; IQR, interquartile range; SD, standard deviation.

## Data Availability

The datasets generated during and/or analysed during the current study are available from the corresponding author on reasonable request.

## Supplementary Materials

The following supporting information can be downloaded at: https://www.mdpi.com/article/10.3390/ijms23031619/s1. Reference [39] is cited in the supplementary materials.
