# Supplementary material for: PD-L1 Expression in Monocytes Correlates with Bacterial Burden and Treatment Outcomes in Active Pulmonary Tuberculosis
Source: Int J Mol Sci. 2022 Jan 30;23(3):1619. doi: 10.3390/ijms23031619 (PMC8836118; doi:10.3390/ijms23031619)
Supplement: Supplementary file 1 [file ijms-23-01619-s001.zip › ijms-1562355-supplementary.pdf]

Methods

Patients and settings

The status of LTBI in non-TB cases was determined with whole-blood-based QuantiFERON-TB Gold In-Tube (QFT-GIT; Qiagen; Germantown, MD, USA) as previously prescribed [1]. Exclusion criteria included active TB patients under anti-TB treatment for ≥ 7 days, active TB patients without pulmonary involvement, subjects <20 years of age, and individuals with active malignancies, human immunodeficiency virus infection, active autoimmune diseases, and organ transplants.

Expression of PD-1 and PD-L1 on peripheral blood mononuclear cells (PBMCs)

PBMCs collected from participants were isolated by Ficoll density-gradient centrifugation were suspended in RPMI-1640 medium (Thermo Fisher Scientific, Waltham, MA, USA) supplemented with 10% fetal bovine serum (Gibco, Carlsbad, CA, USA) and 1% Pen Strep (Thermo Fisher Scientific). The following fluorescently-conjugated human monoclonal antibodies were used: anti-CD4-APC-H7 (clone RPA-T4), anti-CD8-FITC (clone RPA-T8), anti-PD1-PERCP-Cy5.5 (clone EH12.1), anti-PD-L1-PE-Cy7 (clone M1H1), and anti-CD14-APC-H7 (clone MφP9) (BD Biosciences, San Jose, CA, USA).

Treatment outcomes of active TB patients

All enrolled patients with active TB started anti-TB treatment with a standard

regimen that included isoniazid, rifampin, ethambutol, and pyrazinamide. The dosages of the anti-TB drugs were adjusted based on patient weight and renal function. The regimen was modified according to the results of drug susceptibility tests or when significant adverse reactions occurred. All patients were treated for at least 6 months. The treatment outcomes analyzed in the present study included 1- and 2-month smear/culture conversion. Sputum smear/culture conversion was defined as the absence of MTB in smears or cultures of all sputum collected 1 or 2 months after initiation of anti-TB treatment, and the lack of positive cultures thereafter until the completion of anti-TB treatment.

Preparation of human and mouse macrophages

For in vitro studies, THP-1 human leukemia cells (TIB202; ATCC; Manassas, VA, USA) were cultured in RPMI 1640 medium at a density of 1×10^6 cells/mL. The cells were differentiated into macrophages using 20 ng phorbol 12-myristate 13-acetate (PMA, P8139; Sigma-Aldrich; St. Louis, MO, USA) for 24 h followed by a 72-h rest before treatment with MTB-related stimulants. Bone marrow derived macrophages (BMDMs) from the femur and tibia of 8- to 12-week-old male wild-type C57BL/6J mice were cultured for 5 days at a density of 1×10^6 cells/mL in RPMI 1640 medium supplemented with 10% fetal bovine serum, 1% Pen Strep, and 20 ng/mL of cell line-produced granulocyte-macrophage colony-stimulating factor (GM-CSF; R&D

Systems; Minneapolis, MN, USA) at 37 °C in a humidified atmosphere containing 5% CO_2. On days 3 and 5, floating cells were discarded, and fresh medium containing 20 ng/mL GM-CSF was added. On day 6, attached cells were collected by washing with cold 5 mM EDTA in Dulbecco's phosphate buffered saline (PBS). The BMDMs were plated in 24-well microculture plates at a density of 5×10^5 cells/well for 24 h before stimulation.

MTB-related materials and in vitro stimulation

For MTB whole cell lysates preparation, MTB cells were suspended in PBS and disrupted using a French press (approximately 90% breakage). The lysate was centrifuged to pellet the unbroken cells. The whole cell lysate contained the proteins, lipids, and carbohydrates of the bacteria. The recombinant EsxA and CFP-10 proteins were expressed in *Escherichia coli* BL21 (DE3) pLysS and purified by immobilized metal affinity chromatography. PMA-treated THP-1 cells and BMDMs were plated in 24-well microculture plates (1×10^6 and 5×10^5 cells/well, respectively) before in vitro stimulation.

Quantitative reverse transcription PCR and western blot

Total RNA was extracted from the treated THP-1 cells and BMDMs using TRIzol reagent (Invitrogen; Carlsbad, CA, USA). Quantitative reverse transcription PCR was performed in duplicate with the Power SYBR green master mix (Roche; Mannheim,

Germany) and analyzed with the Mx3000P real-time PCR system (Stratagene; San Diego, CA, USA). The relative expression of *PD-L1* mRNA was measured and normalized to glyceraldehyde 3-phosphate dehydrogenase. Primer sequences were human PD-L1 (sense) TGGCATTTGCTGAACGCATT, (anti-sense) TGCAGCCAGGTCTAATTGTTTT; mouse PD-L1 (sense) TGGCATTTGCTGAACGCATTT , (anti-sense) TGCAGCCAGGTCTAATTGTTTT.

THP-1 cells and BMDMs were harvested after stimulation and lysed in lysis buffer. Equal amounts of proteins were separated by SDS-PAGE and electrotransferred to PVDF membranes. After incubation with primary antibodies to PD-L1, the blots were visualized using an enhanced chemiluminescence western blot detection system.

IHC and IF analysis

To identify PD-1 and PD-L1 expressing cells, lung specimens obtained from two active pulmonary TB patients analyzed by IHC. Briefly, 4 μm sections of lung tissues were pre-treated using antigen retrieval solution and incubated with anti-PD-1 antibody (Abcam Inc., Cambridge, UK) and anti-PD-L1 antibody (R&D Systems) at 10 mg/mL (diluted 1:100) for 60 min at room temperature. The expression of antibodies was detected using a horseradish peroxidase-conjugated compact polymer system. IHC staining was performed using anti-CD68 antibody (Dako; Glostrup, Denmark) to identify macrophages in the lung tissues. IHC analysis was performed with the Leica

Bond-MAX automatic IHC staining system. IF was performed to evaluate PD-1 and PD-L1 expression in lung specimens from mice following intratracheal instillation of HKMTB. To obtain lung tissue, perfusion was performed by injecting 5 mL cold PBS through the right ventricle of the heart before embedding. Lung tissues were fixed in 3.7% formalin overnight, cryoprotected in 30% sucrose/PBS overnight, and then cryoembedded in O.C.T. Lung sections (4 µm) were stained with primary antibodies against PD-1 (Cell Signaling Technology, Beverly, MA, USA), PD-L1 (GeneTex; Irvine, CA, USA), CD3 (Abcam), and F4/80 (Abcam). Alexa Fluor conjugates (Invitrogen) were used as secondary antibodies. Nuclear staining was achieved using 4',6-diamidino-2-phenylindole (DAPI). Negative control experiments in the absence of primary antibodies were performed to indicate the level of background autofluorescence. Fluorescence images were obtained using 3D HISTECH PANNORAMIC SCAN and panoramic viewer software.

Reference

1. Feng, J.Y.; Huang, S.F.; Lee, M.C.; Ting, W.Y.; Chen, Y.C.; Lin, Y.Y.; Lee, Y.C.; Su, W.J. Characteristics of IFN-gamma responses in IGRA among pulmonary TB suspects in a TB-endemic area. *Diagn. Microbiol. Infect. Dis.* **2013**, *77*, 46–52.
